# Quantitative proteomic analysis reveals different characteristics of bladder cancer cells after exposure to bisphenol A

**DOI:** 10.1002/2211-5463.70221

**Published:** 2026-02-25

**Authors:** Shaomin Niu, Lanlan Li, Wenjuan Jia, Hanzhong Feng, Xiaoquan Yu, Youli Zhao, Fengzhi Yuan, Hui Ding, Junqiang Tian, Yong‐Xing He, Zhiping Wang

**Affiliations:** ^1^ Institute of Urology, Clinical Research Center for Urology in Gansu Province, The Second Hospital & Clinical Medical School Lanzhou University Gansu China; ^2^ Institute of Otolaryngology‐Head & Neck Surgery, The Second Hospital & Clinical Medical School Lanzhou University Gansu China; ^3^ Ministry of Education Key Laboratory of Cell Activities and Stress Adaptations, School of Life Sciences Lanzhou University Gansu China; ^4^ Department of Clinical Laboratory The Second Hospital of Lanzhou University Gansu China

**Keywords:** Bisphenol A, Bladder cancer, inflammation response, MAPK signal, Proteomics

## Abstract

Bisphenol A (BPA) is a widely used industrial chemical present in numerous consumer products such as plastics, epoxy resins, and thermal paper. Growing evidence suggests that BPA may pose potential health risks, particularly in relation to carcinogenesis. In this study, we investigated the toxic effects and carcinogenic role of BPA using two bladder cancer cell lines, T24 and UMUC. Our results show that BPA influences cell viability and migration in a dose‐dependent manner. At low concentrations, BPA significantly promoted cell growth and cell migration in both T24 and UMUC cells, whereas higher concentrations suppressed cell growth. Proteomic and transcriptomic analyses further revealed substantial changes in protein and gene expression profiles following BPA exposure. Bioinformatics analysis suggested that BPA may modulate bladder cancer cell behavior through the MAPK signaling and inflammatory response pathways. Together, these findings offer important insights into the molecular mechanisms of BPA‐induced cellular alterations, with potential implications for developing more targeted and effective therapeutic strategies for cancer. Future studies should focus on further elucidating the signaling pathways affected by BPA, exploring its potential synergistic or antagonistic interactions with other environmental factors, and validating these results through *in vivo* models.

## Graphical abstract

Bisphenol A (BPA), a common chemical in plastics, exerts dual effects on bladder cancer cells: low doses promote growth and migration, while high doses suppress growth and migration. Multi‐omics and bioinformatics reveal BPA acts via MAPK and inflammatory pathways. These insights into BPA‐induced molecular changes inform potential cancer therapeutic strategies.

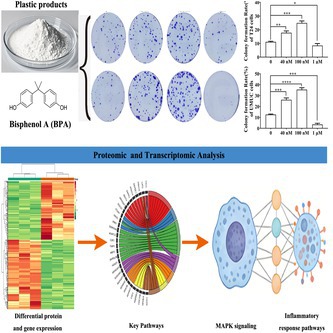

**Abbreviations**
4D‐DIA: four‐dimensional‐Data Independent Acquisition
AR: androgen receptor
BC: bladder cancer
BCA: bicinchoninic acid
BPA: Bisphenol A
CCK8: cell counting kit‐8
DTT: dithiothreitol
ER: estrogen receptor
GO: gene ontology
IAA: iodoacetamide
KEGG: Kyoto Encyclopedia of Genes and Genomes
LC–MS/MS: Liquid chromatography–tandem mass spectrometry
MAPK: Mitogen‐Activated Protein Kinase
PCA: principal components analysis
THR: thyroid hormone receptor

Bisphenol A (BPA) is a widely used environmental endocrine disruptor employed in the production of various plastic products, such as polycarbonate, epoxy resins, and other polymer materials [1]. It can be found in many daily necessities, causing serious environmental pollution and posing a great threat to human health [2, 3]. In surface waters, BPA is frequently detected at concentrations typically ranging up to the nmol/L level in river systems [4]. Although BPA is a common contaminant and has been detected in Chinese drinking water, its concentrations are generally low, often around 12.8 ng·L^−1^ [5]. Human exposure to BPA is thought to occur mainly through the migration of BPA from dietary packaging materials into food. However, other exposure routes also exist, such as inhalation of indoor dust and dermal contact. Pharmacokinetic studies have shown that BPA is distributed in almost all the human tissues, with the highest concentration in adipose tissue, followed by liver and brain [6], serum [7], urine [8], semen [9], and maternal serum including fetal serum and term amniotic fluid [10, 11]. Studies have revealed that BPA exhibits various health hazards to the development of cancer, heart disease, diabetes, hypertension, male reproduction abnormality, and obesity [12].

BPA is known to interfere with the signaling pathways of various nuclear receptors, including the estrogen receptor (ER), androgen receptor (AR), and thyroid hormone receptor (THR) [13]. Increasing evidence indicates that BPA exposure increases the risk of hormone‐dependent cancers, including those of the prostate, breast, and ovary [14, 15, 16]. In prostate cancer, for instance, BPA activates a tumor‐derived AR mutant (T877A), promoting androgen‐independent proliferation of cancer cells. These findings support the concept that nonsteroidal environmental compounds can modulate the function of nuclear receptor complexes [17]. In addition, BPA can also exhibit toxicity to hormone‐independent tissue, such as bladder and kidney tissues. BPA is detectable in over 90% of human urine samples [18], reflecting widespread and continuous exposure. Although BPA has been shown to promote bladder cancer progression by altering the energy metabolism of stromal cells [19], its specific role in regulating cellular behavior and molecular mechanisms across different stages of bladder cancer remains poorly understood.

Notably, bladder cancer (BC) ranks as the 10th most commonly diagnosed cancer worldwide, with an estimated age‐standardized incidence rate of approximately 9.5 per 100 000 and a mortality rate of about 3.3 per 100 000 [20]. In China, the incidence and mortality rates are relatively lower yet remain substantial, reported at around 7.0 and 2.7 per 100 000, respectively [21]. The disease exhibits a strong age dependence, with the majority of cases occurring in individuals over 65 years of age, and men are affected approximately 3–4 times more frequently than women [22]. In the urinary system, oral exposure to BPA, even for a short duration, has been shown to impact the intramural neurons within the urinary bladder wall. Alterations in the neurochemical profile of these neurons may serve as the initial indicators of BPA‐induced pathological processes in this organ [23]. In addition, chronic exposure to BPA not only decreases the energy metabolism and properties of urothelial cells but also enhances these properties in bladder cancer cells, ultimately promoting bladder cancer progression [24, 25]. However, the effects of BPA exposure on protein and transcript expression profiles in bladder cancer cells are still relatively limited.

In this study, we found that low concentration of BPA could promote cell viability and migration of bladder cancer cells at different stages. Through a comprehensive approach integrating proteomic and transcriptomic analyses, our investigation unveiled that BPA likely exerts its influence on cellular behaviors through the Mitogen‐Activated Protein Kinase (MAPK) signal and inflammation response pathway.

## Materials and methods

### Cell culture and treatment

Two human BC cell lines UMUC‐3 (TCHu217) and T24 (SCSP‐536) were purchased from the Chinese Academy of Sciences Cell Bank (Shanghai, China). BC cells were cultured in RPMI‐1640 medium (L210KJ, BasalMedia, Shanghai, China) containing 7% fetal bovine serum (FSP500, Excell Bio, Shanghai, China) in an incubator with 5% CO_2_ at about 37 °C. Bladder cancer cells T24 and UMUC were treated for 48 h with or without BPA. BPA (CAS 80‐05‐7, purity ≥ 99%) was purchased from Beijing Bailingwei Technology Co., Ltd (Beijing, China) and primarily dissolved in DMSO. All cell lines have been authenticated by Fuheng Biology (China) within the past 3 years. DNA was extracted using the Axygen genomic DNA extraction kit, amplified with a 20‐STR amplification protocol, and detected for STR loci and the gender‐determining gene Amelogenin on an ABI 3730XL genetic analyzer. All cell lines also were confirmed to be free of mycoplasma contamination.

### Cell viability

Cell viability was determined by cell counting kit‐8 (CCK8; K1018, ApexBio, Beijing, China) assay. About 4.0 × 10^3^ cells per well of each cell line were seeded in parallel in two 96‐well plates and incubated at 37 °C with CO_2_ for 24 h. Subsequently, the cells were exposed to 0.1% DMSO or different concentrations of BPA (10 nm, 40 nm, 100 nm, 1000 nm, 10 000 nm) for 48 h. Then, CCK8 solution was added into each well and incubated for 2–4 h at 37 °C in the dark. Finally, the OD of each cell line was determined using a chemiluminescence detector at a wavelength of 450 nm. The cell viability was normalized to the OD of 0 nm BPA (0.1% DMSO). Each experiment was independently repeated three times (*n* = 3).

### Colony formation assay

Cells were seeded in a six‐well plate with a density of 400 cells per well and treated with BPA at various concentrations (0 nm, 40 nm, 100 nm and 1 μm) for 48 h. Then, the cell culture medium was replaced every 3 days, and the cells were cultured for 10–14 days. Following the incubation period, cells were fixed with 4% paraformaldehyde and stained with 0.1% crystal violet solution. Colonies were imaged, and the colony count was quantified using the ImageJ software (NIH, Bethesda, MD, USA). The colony formation rate was calculated as (number of colonies/number of seeded cells) × 100%.

### Scratch tests

UMUC and T24 cells were seeded in six‐well culture plates at a density of 5 × 10^5^ cells per well. The cells were allowed to adhere, and then a straight line across the well was made using a 200 μL pipette tip. After washing with PBS, the cells were treated with BPA at concentrations of 0 (control), 40 nm, 100 nm, and 1 μm for 48 h. Cell migration in each well was captured under a light microscope. The cell migration rate was calculated using the following formula: (scratch width_0h_ – scratch width_48h_)/scratch width_0h_.

### Four‐dimensional‐data independent acquisition (4D‐DIA) quantitative proteome analysis

Protein was extracted and lysed from the cell sample and the supernatant was collected and subjected to quantification with bicinchoninic acid (BCA) assay. Then, the equivalent protein was treated with dithiothreitol (DTT) and iodoacetamide (IAA) to inhibit the activity of reduced cysteine and precooled acetone was used to precipitate protein. The precipitated protein was resuspended and subjected to digestion with trypsin overnight at 37 °C and subsequent collecting and desalting using a C18 StageTip in preparation for Liquid chromatography–tandem mass spectrometry (LC–MS/MS) analysis. LC–MS/MS analysis was conducted using a timsTOF Pro2 mass spectrometer, which was connected with a NanoElute UHPLC system. The obtained raw files were searched against uniport_proteome UP000005640_human_20230504 fasta database (82 492 total entries) using DIA‐NN (v1.8.1). The threshold for differential protein analysis was a fold change greater than 1.2 and a *P*‐value less than 0.05.

### Transcriptome sequencing analysis

Total RNA was extracted from the cell sample and RNA integrity was measured. The extracted RNA was fragmented into smaller pieces, subjected to cDNA synthesis, library preparation, and sequencing using the Illumina platform. The raw sequencing data are processed through a series of bioinformatics analyses to remove sequencing artifacts, align the reads to a reference genome or transcriptome, quantify gene expression levels, identify alternative splicing events, and perform other transcriptomic analyses. The threshold for differential gene analysis was a fold change greater than 2 and a *P*‐value less than 0.05.

### Bioinformatics analysis

Transcriptome and proteome data were analyzed with Bioladder platform (https://www.bioladder.cn/web/#/firstVue) that is based on R language, DAVID (https://david.ncifcrf.gov/), and the analysis results are presented using Graphpad Prism 8.0.

### Statistical analysis

Comparisons between two groups were analyzed using unpaired two‐tailed Student's *t*‐test. Comparisons between three or more groups were analyzed using one‐ or two‐way analysis of variance (ANOVA) with Graphpad Prism 8.0. Statistical significance is indicated as follows: *P < 0.05; **P < 0.01; ***P < 0.001; ****P < 0.0001.

### Data availability

The mass spectrometry proteomics data have been deposited to the ProteomeXchange Consortium via the PRIDE. Partner repository with the dataset identifier PXD060021.

## Results and discussion

### BPA affected cell viability in a dose‐dependent manner

**Fig. 1:**
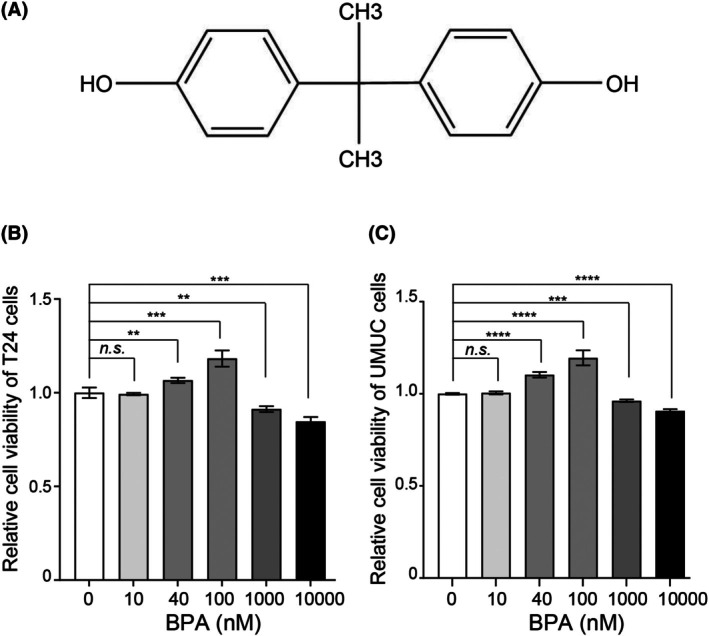
BPA affected cell viability in a dose‐dependent manner in T24 and UMUC cells. (A) Chemical structures of BPA. (B, C). Cell viability of T24 cells (B) and UMUC cells (C) was determined after cells were exposed to different doses of BPA for 48 h. The data are presented as mean ± SD. All experiments were repeat three times. An unpaired *t*‐test was used, *P < 0.05; **P < 0.01; ***P < 0.001; ****P < 0.0001.

T24 cells and UMUC cells are the common urinary bladder cancer cells and are usually used in cancer research. The T24 cell line exhibits relatively high invasiveness and aggressiveness, along with a rapid cell proliferation rate. In contrast, UMUC cell lines display a slower cell proliferation rate [26, 27]. To examine the impact of BPA (Fig. 1A) on T24 and UMUC cells, BPA was added into the cell culture, followed by a CCK8 assay to evaluate cell viability. As shown in Fig. 1B,C, BPA promoted viability at low concentrations (40 nm and 100 nm) but inhibited cell viability at high concentrations (1000 nm and 10 000 nm), suggesting that BPA can affect cell viability in a dose‐dependent manner. High concentrations of BPA (> 100 μm) have been found to reduce the cell viability of human 3 T3‐L1 and KGN cells, and goat testis sertoli cells, respectively [28, 29, 30]. Given that 10 000 nm (10 μm) of BPA also reduces cell viability in both T24 and UMUC cells, the impact of high concentrations of BPA on bladder cancer cell viability appears to be similar to that observed in other cell types.

### Low concentration of BPA promoted cell migration and colony formation

**Fig. 2:**
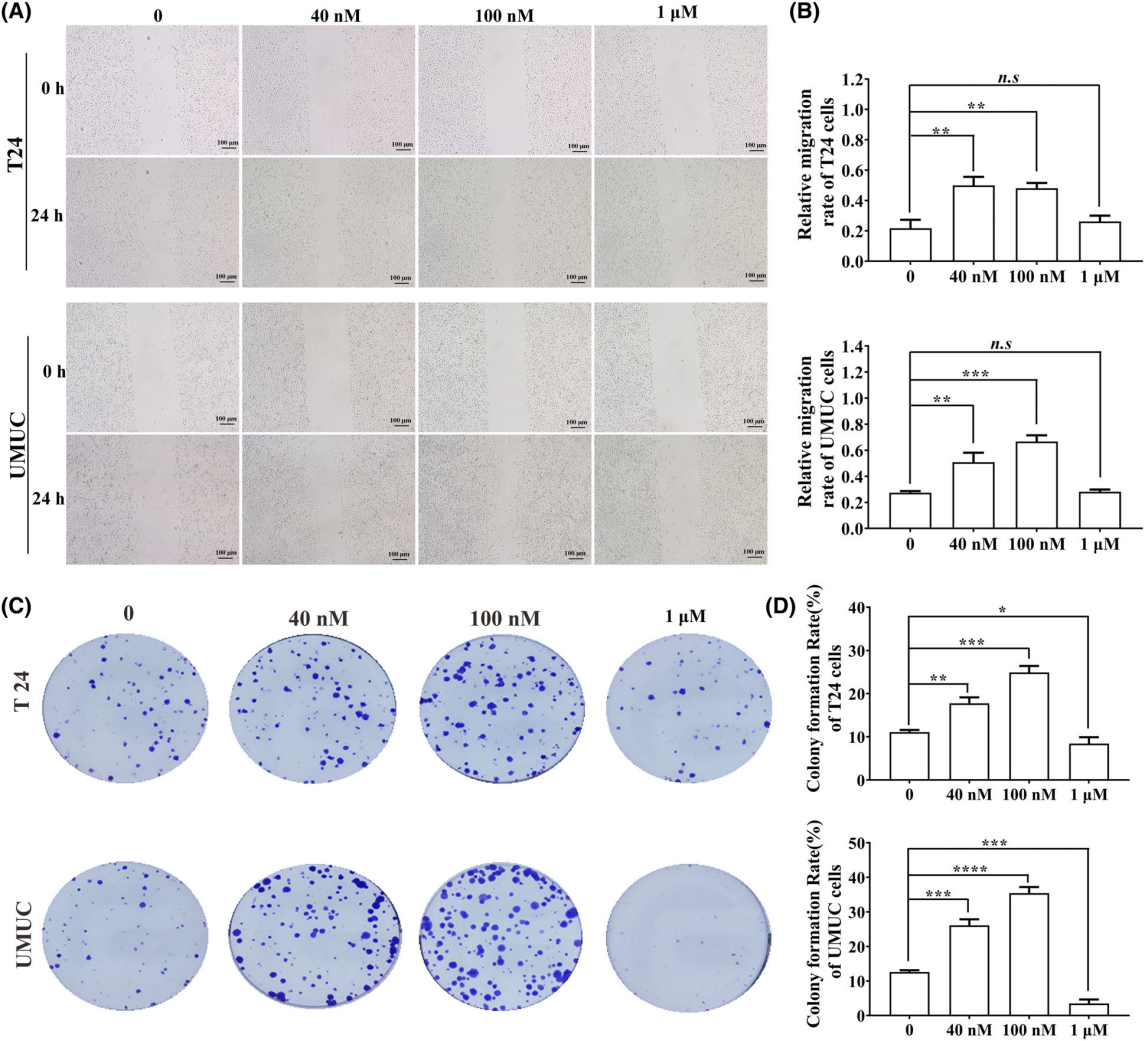
Low concentration of BPA promoted cell migration colonies formation of T24 and UMUC cells. (A) Scratch test of T24 and UMUC cells after cells were exposed to different doses of BPA for 0 and 48 h, respectively. (B) Statistical analysis of cell migration in T24 and UMUC cells. (C) Colony formation of T24 and UMUC cells treated with low concentration of BPA. (D) Statistical analysis of colony formation rates in T24 and UMUC cells. The data are presented as mean ± SD. All experiments were repeated three times. An unpaired *t*‐test was used, *P < 0.05; **P < 0.01; ***P < 0.001; ****P < 0.0001.

In addition, we also assessed the effect of BPA on cell migration using a wound healing assay and evaluated its impact on clonogenic ability with a colony formation assay. Low concentrations of BPA (40 nm and 100 nm) significantly promoted cell migration and colony formation in both T24 and UMUC cells, whereas a high concentration of BPA (1 μm) inhibited cell migration and colony formation (Fig. 2A–D). Bin Jia *et al*. [31] have found that 10 nm of BPA exposure induces Hela and HepG2 cell migration via activating directly integrin β1. BPA also stimulates A549 cell migration via upregulation of matrix metalloproteinases by the GPER/EGFR/ERK1/signal pathway [16]. These findings indicate that exposure to BPA can facilitate cancer cell migration. Notably, low concentrations of BPA have the opposite effect to high concentrations. Considering that BPA is commonly detected in surface waters, with concentrations in the range of nmol L^−1^ in river water, our findings indicate that low levels of BPA enhance cell viability and migration, both of which are biological processes associated with cancer progression. Consequently, it is crucial to be aware of the connection between exposure to BPA in domestic water and the rising incidence of cancers.

### Proteomic profiling of T24 and UMUC cells after exposure to BPA

**Fig. 3:**
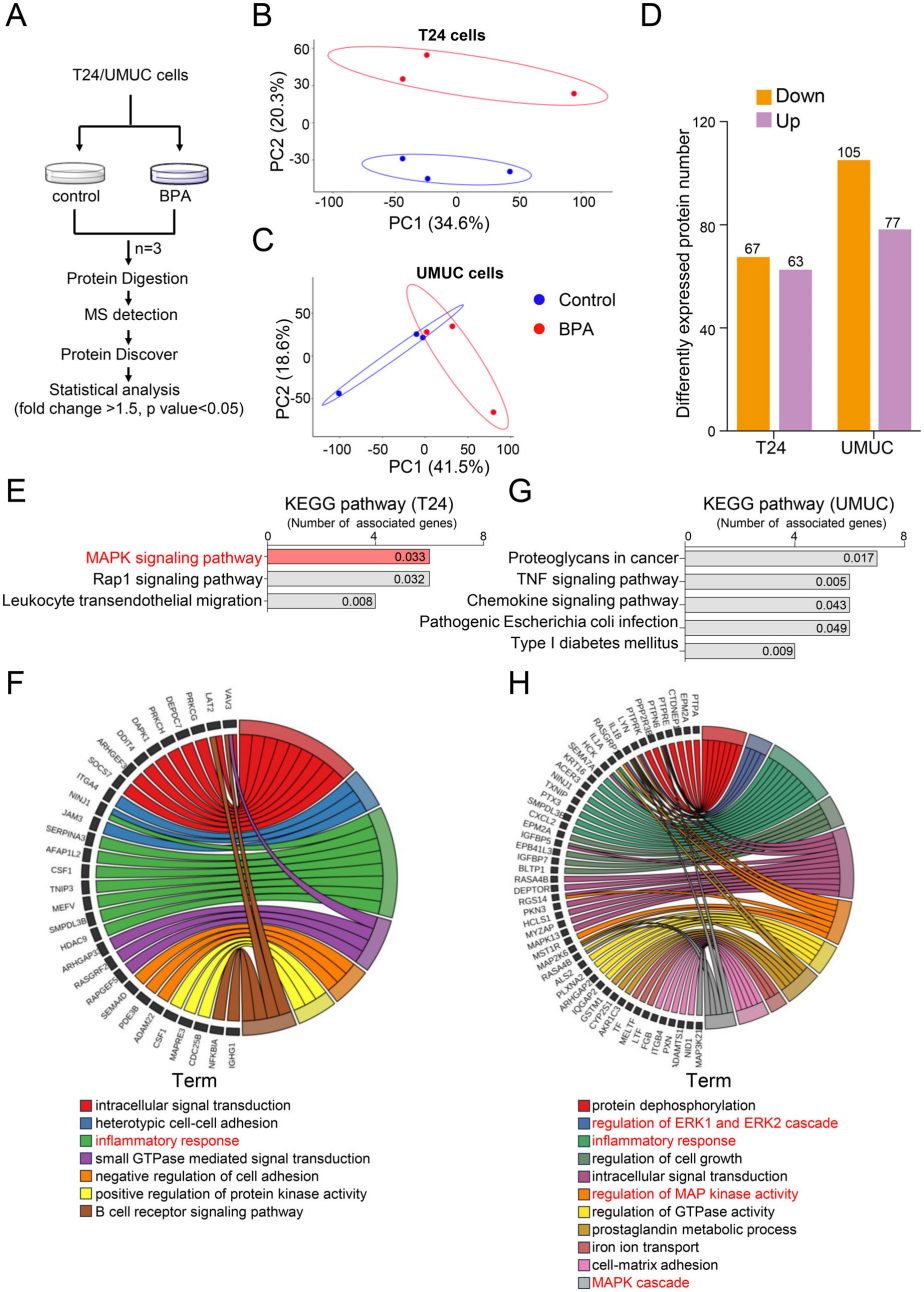
The proteomic analysis of T24 and UMUC cells exposed to low concentrations of BPA. (A) Experimental strategy to characterize proteomic changes. (B, C) The principal component analysis (PCA) chart of BPA group and control group in T24 cells (B) and UMUC cells (C). (D) Histogram statistics of differential protein in T24 and UMUC cells after exposure to BPA. (E, F) KEGG (E) and GO (F) analysis of differential protein in T24 cells. (G, H) KEGG (G) and GO (H) analysis of differential protein in UMUC cells.

To further investigate the effect of BPA on the regulation of T24 and UMUC cells, both cell lines were cultured and subsequently subjected to proteomic analysis (Fig. 3A). Principal component analysis (PCA) demonstrated a clear separation between the proteome profiles of samples in the BPA group and those in the control group for T24 cells (Fig. 3B). However, the impact of BPA on UMUC cells appeared to be less pronounced compared to its effect on T24 cells. The PCA indicated that the BPA group in UMUC cells did not separate completely from the control group (Fig. 3C). Based on a fold‐change greater than 1.2 and *P*‐value less than 0.05 between the two groups, we identified 130 significantly differentially expressed proteins in the BPA group compared to the control group in T24 cells, and 182 in UMUC cells (Fig. 3D) (Table S1). Kyoto Encyclopedia of Genes and Genomes (KEGG) and Gene Ontology (GO) analysis were performed to gain insights into the biological functions of those differentially expressed proteins. The bioinformatics enrichment analysis showed that in T24 cells, exposure to BPA may influence the MAPK signaling pathway, Rap1 signaling pathway, intracellular signal transduction, heterotypic cell–cell adhesion, and inflammatory response (Fig. 3E,F). Interestingly, similar biological pathways, such as proteoglycans in cancer, TNF signaling pathway, protein dephosphorylation, regulation of ERK1 and ERK2 cascade, inflammatory response, regulation of cell growth, intracellular signal transduction, and regulation of MAP kinase activity, were also enriched in the UMUC cells (Fig. 3G,H). The complete proteomic raw data from all KEGG pathway and GO enrichment analyses are provided in Tables S2 and S3.

**Fig. 4:**
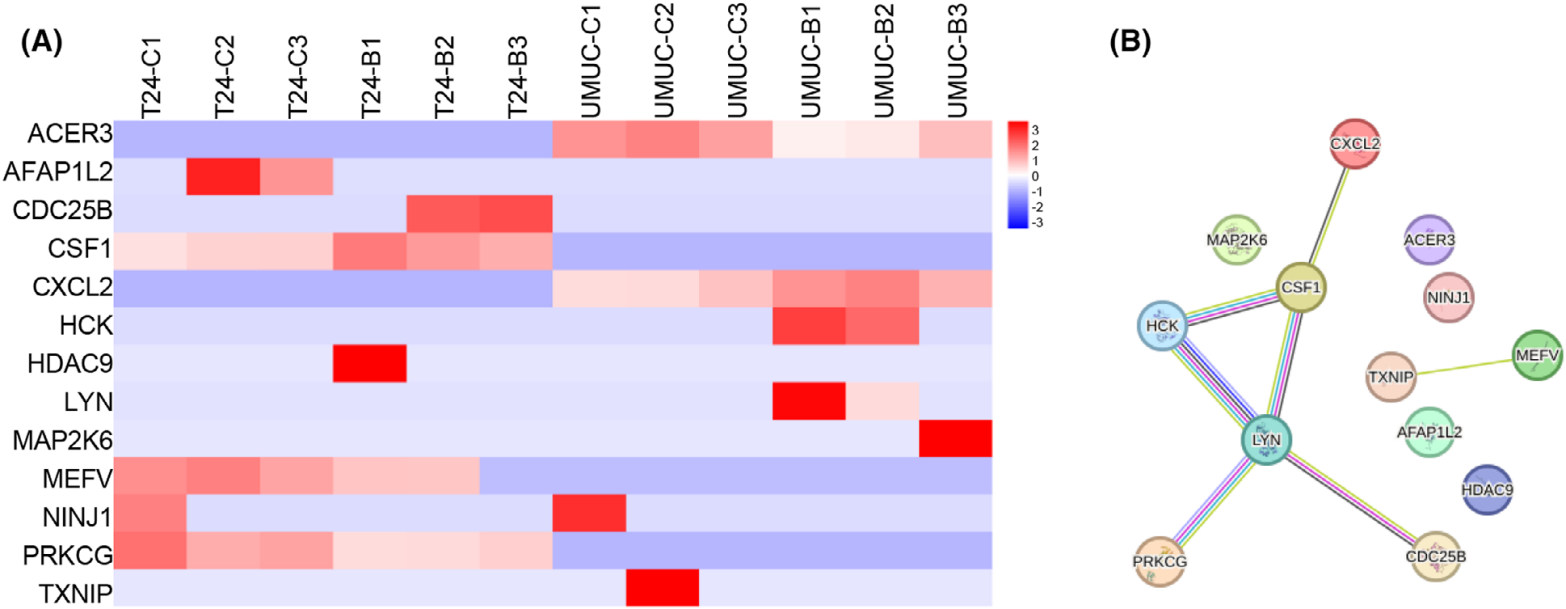
BPA regulates the MAPK signal and inflammation response pathways in T24 and UMUC cells through the modulation of different proteins. Heatmap (A) and protein–protein interaction network (B) of proteins enriched in MAPK signal pathway, inflammation response pathways, and other related pathways enriched in T24 and UMUC cells. Plots of protein–protein interactions were generated from the STRING web tool (https://cn.string‐db.org/).

Given the enrichment of the MAPK signaling pathway, inflammatory response, and their related pathways in both T24 and UMUC cells, we further focused on the expression patterns of proteins associated with these pathways in different samples. As shown in Fig. 4A,B, the proteins related to these pathways are not identical in T24 and UMUC cells, and there is a significant differential expression pattern between T24 and UMUC cells, suggesting BPA may regulate the MAPK pathway, inflammatory response, and their related pathways through the modulation of distinct genes in different bladder cancer cells.

### Transcriptomic analysis of T24 and UMUC cells treated with BPA

**Fig. 5:**
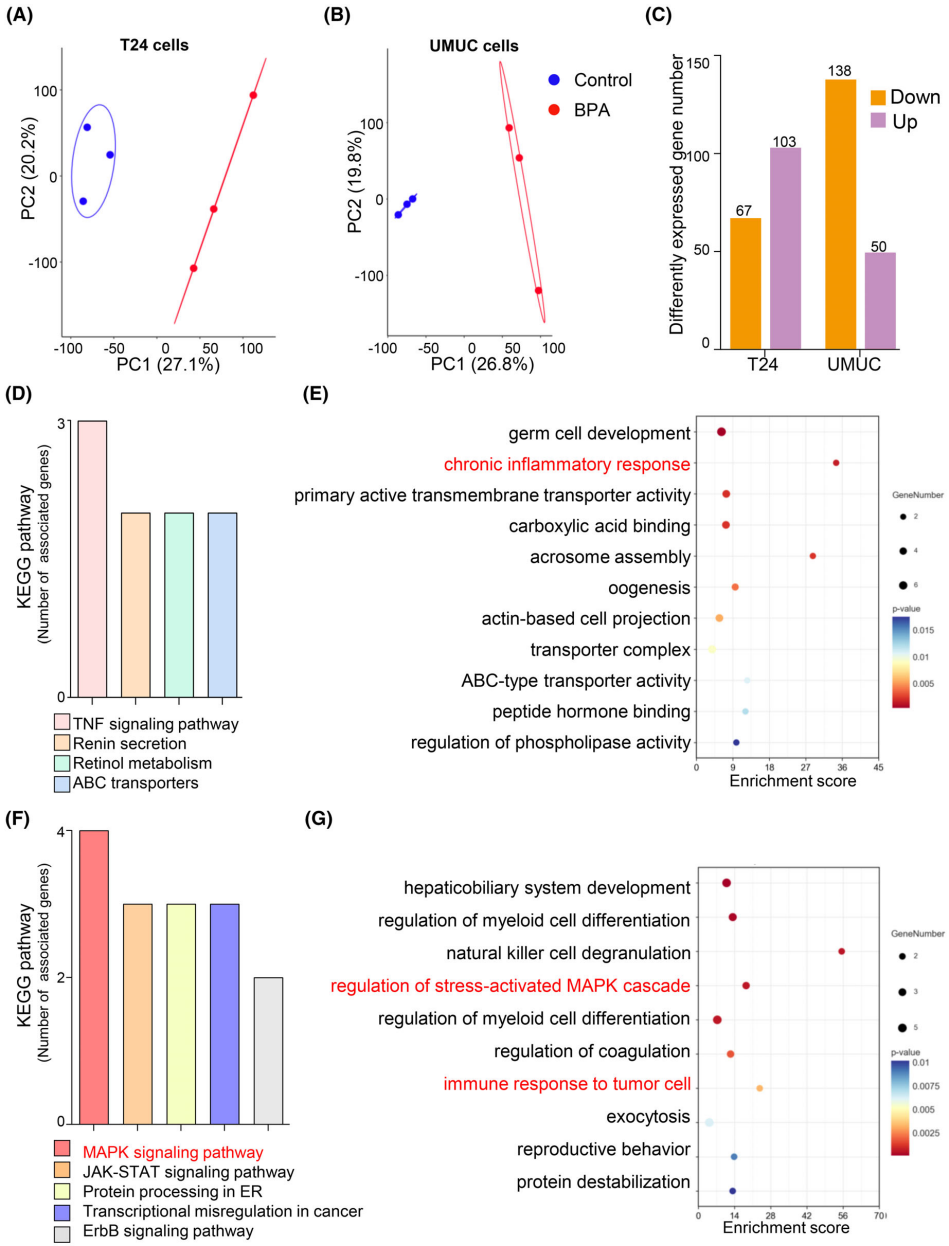
The transcriptomics analysis of T24 and UMUC cells treated with 100 nm BPA. (A, B) PCA analysis of BPA group and control group in T24 cells (A) and UMUC cells (B). (C) Differential genes in T24 and UMUC cells after exposure to BPA. (D–G) Signaling pathways involved by altered expression of genes in BPA group with KEGG (D, F) and GO analysis (E, G) in both T24 (D, E) and UMUC (F, G) cells.

We also performed transcriptomic analysis to identify the effects of BPA on the transcriptional levels of T24 and UMUC cells. Principal components analysis (PCA) showed a clear separation between the transcriptional profiles of samples in the BPA group and those in the control group for both T24 and UMUC cells (Fig. 5A,B). According to a fold‐change greater than 2 and a *P*‐value less than 0.05, we found 170 genes that were significantly differentially expressed in the BPA group compared to the control group in T24 cells, and 188 genes in UMUC cells (Fig. 5C). Intriguingly, the KEGG and GO enrichment results of the transcriptome exhibited similarities to those of the proteome.

In T24 cells, the TNF signaling pathway, germ cell development, and chronic inflammatory response were enriched following exposure to BPA (Fig. 5D,E). In UMUC cells, enrichment was observed in the MAPK signaling pathway, hepaticobiliary system development, and immune response to tumor cells (Fig. 5F,G). The complete transcriptomic raw data from all KEGG pathway and GO enrichment analyses are provided in Tables S4 and S5. Those results from the proteomic and transcriptomic analyses indicate that BPA regulates the biological processes of bladder cancer cells through modulation of the MAPK pathway and the inflammatory response.

The MAPK signal cascade, consisting of RAF, MEK, and ERK, has been found to direct cellular responses to a variety of stimuli, including inflammatory cytokines (such as TNF‐α and IL‐6), osmotic stress, and heat shock [32]. It is involved in cell proliferation, differentiation, survival, migration, and invasion, and plays a crucial role in tumor initiation, progression, and development [33, 34]. In bladder cancer, activation of the MAPK pathway could positively promote cell viability and proliferation [32, 35]. Combinatorial inhibition of the MAPK pathway with other signaling pathways, such as NOTCH, can effectively suppress bladder tumor growth [36]. In addition, inflammation serves as a cancer risk factor by initiating tumorigenesis through the induction of DNA damage, even in the absence of any exogenous carcinogens [37]. Chronic inflammation, induced by infections, aberrant immune reactions, or environmental factors such as environmental contaminants, significantly increases the risk of tumor development [37]. Bladder cancer is often linked to chronic or recurrent inflammation, with a significant presence of inflammatory cells commonly observed at the tumor site [38]. In addition to its reported effects on normal urothelial and bladder cancer cells, BPA has been shown to impact bladder cancer recurrence, progression, and patient prognosis [24]. Our findings further provide evidence that BPA may influence these cellular behaviors through modulating the MAPK signaling pathway and inflammation response.

## Conclusion

In conclusion, this study provides valuable insights into the proteome and transcriptome changes induced by BPA in different bladder cancer cells. The findings demonstrate the diverse and multifaceted impact of BPA on various cellular processes and pathways, contributing to our understanding of its role in tumorigenesis. Our data suggest that in both T24 and UMUC cells, BPA affects the MAPK signaling pathway, inflammatory response, intracellular signal transduction, cell–cell adhesion, and TNF signaling pathway, with implications for cancer cell proliferation, migration, and invasion. These findings highlight the significance of comprehending the multifaceted effect of BPA in various stages of bladder cancer cells. Furthermore, they serve as a reminder for us to exercise caution regarding the potential of low concentrations of BPA in promoting cancer progression in the environment.

By elucidating the molecular mechanisms underlying BPA‐induced cellular changes, this study contributes to the development of more targeted and effective preventive and therapeutic strategies for cancer patients. Future research should focus on further dissecting the molecular mechanisms and signaling pathways modulated by BPA, as well as investigating potential synergistic or antagonistic effects with other environmental factors. Additionally, *in vivo* studies will be crucial in validating the findings from this study and assessing the clinical relevance of these proteome changes in the context of cancer development and progression.

In summary, our study bridges the gap between environmental BPA exposure and bladder cancer progression by uncovering the underlying proteomic mechanisms. These findings contribute to public health by reinforcing BPA's carcinogenic potential for bladder cancer, enabling targeted surveillance and prevention, and providing mechanistic evidence to inform stricter risk evaluation and regulation. Ultimately, our work supports a more comprehensive approach to mitigating BPA‐related health risks, particularly for vulnerable populations, and guides future research to translate molecular insights into actionable public health interventions.

## Conflict of interest

The authors declare no conflict of interest.

## Author contributions

SN was involved in conceptualization, methodology, data curation, formal analysis, writing – original draft. LL was involved in conceptualization, methodology, formal analysis, writing – original draft, funding acquisition. WJ was involved in conceptualization, methodology, formal analysis. HF, YZ, and XY were involved in methodology. FY, HD, and JT were involved in conceptualization. HY was involved in conceptualization, writing – review and editing, supervision. ZW was involved in conceptualization, writing – review and editing, funding acquisition, supervision.

## Supporting information


**Table S1.** Significantly differentially expressed proteins in the BPA group compared to the control group in T24 cells (130 proteins) and UMUC cells (182 proteins).
**Table S2.** The full set of KEGG pathway and GO enrichment analysis results generated from the proteomic profiling of T24 cells.
**Table S3.** The full set of KEGG pathway and GO enrichment analysis results generated from the proteomic profiling of UMUC cells.
**Table S4.** Significantly enriched signaling pathways in GO analysis of T24 cells and UMUC cells.
**Table S5.** The full set of KEGG pathway and GO enrichment analysis results generated from the transcriptomic profiling of T24 cells and UMUC cells.

## Data Availability

The data that support the finding of this study are available from the corresponding author upon reasonable request.
